# FOXFIRE protocol: an open-label, randomised, phase III trial of 5-fluorouracil, oxaliplatin and folinic acid (OxMdG) with or without interventional Selective Internal Radiation Therapy (SIRT) as first-line treatment for patients with unresectable liver-only or liver-dominant metastatic colorectal cancer

**DOI:** 10.1186/1471-2407-14-497

**Published:** 2014-07-09

**Authors:** Susan J Dutton, Nicola Kenealy, Sharon B Love, Harpreet S Wasan, Ricky A Sharma

**Affiliations:** 1 Centre for Statistics in Medicine, Nuffield Department of Orthopaedics, Rheumatology and Musculoskeletal Sciences, University of Oxford, Oxford, UK; 2 Oncology Clinical Trials Office, Department of Oncology, University of Oxford, Oxford, UK; 3 Imperial College Healthcare NHS Trust, Hammersmith Hospital, London, UK; 4 CRUK-MRC Oxford Institute for Radiation Oncology, NIHR Biomedical Research Centre Oxford, Department of Oncology, University of Oxford, Old Road Campus Research Building, Oxford, UK

**Keywords:** Colorectal cancer, Liver metastases, Selective Internal Radiation Therapy (SIRT), Radio-embolization, Brachytherapy, Radiosensitisation, Radiosensitization, Quality of life, Health economics

## Abstract

**Background:**

Colorectal cancer (CRC) is the second most common malignancy in Europe and a leading cause of cancer-related death. Almost 50% of patients with CRC develop liver metastases, which heralds a poor prognosis unless metastases can be downsized to surgical resection or ablation. The FOXFIRE trial examines the hypothesis that combining radiosensitising chemotherapy (OxMdG: oxaliplatin, 5-fluorouracil and folic acid) with Selective Internal Radiation Therapy (SIRT or radioembolisation) using yttrium-90 resin microspheres (SIR-Spheres®; Sirtex Medical Limited, North Sydney, Australia) as a first-line treatment for liver-dominant metastatic CRC will improve clinical outcomes when compared to OxMdG chemotherapy alone.

**Methods/Design:**

FOXFIRE is an open-label, multicentre, randomised controlled trial of OxMdG with or without the addition of SIRT (1:1 randomisation). Eligible adult patients have histologically confirmed colorectal adenocarcinoma, liver metastases measurable on computed tomography scan and untreatable by either surgical resection or local ablation, and they may have limited extra-hepatic disease, defined as ≤5 nodules in the lung and/or one other metastatic site which is amenable to future definitive treatment. Eligible patients may have received adjuvant chemotherapy following resection of the primary tumour, but are not permitted to have previously received chemotherapy for metastatic disease, and must have a life expectancy of ≥3 months and a WHO performance status of 0–1. The primary outcome is overall survival. Secondary outcomes include progression free survival (PFS), liver-specific PFS, patient-reported outcomes, safety, response rate, resection rate and cost-effectiveness. FOXFIRE shares a combined statistical analysis plan with an international sister trial called SIRFLOX.

**Discussion:**

This trial is establishing a network of SIRT centres and ‘feeder’ chemotherapy-only centres to standardise the delivery of SIRT across the whole of the UK and to provide greater equity of access to this highly specialised liver-directed therapy. The FOXFIRE trial will establish the potential role of adding SIRT to first-line chemotherapy for unresectable liver metastatic colorectal cancer, and the impact on current treatment paradigms for metastatic CRC.

**Trial registration:**

ISRCTN83867919

## Background

Colorectal cancer is the second most common malignancy in Europe and a leading cause of cancer-related deaths. In the UK, it is the second most common cancer in women after breast cancer, and the third in men after prostate and lung cancer. Despite recent major advances in the systemic treatment of metastatic colorectal cancer (mCRC), the 5-year overall survival (OS) remains disappointingly low at approximately 12% [1]. Median survival for this patient group is currently 1.5-2.5 years, and depends on continuation of chemotherapy for most of a patient’s remaining life. Newly developed, expensive, biologically targeted agents have had marginal effects in improving survival and have not significantly affected the chances of cure [2]. It is notable that in a subset of patients with liver-only mCRC, in whom surgical resection of disease can be achieved, the 5-year survival probability is 27-39%, with 20% of patients achieving long-term cure [3-6].

Liver-directed strategies have been developed with the aim of improving OS in patients with mCRC and liver-only or liver-dominant disease. Selective Internal Radiation Therapy (SIRT or radioembolisation [RE]) is a technique that targets multiple sites of disease within the liver as a form of brachytherapy delivering high doses of ionising radiation directly to the tumour whilst keeping radiation exposure of the normal liver to a tolerable dose [7,8]. SIR-Spheres® (Sirtex Medical Limited, North Sydney, Australia), that contain the β-emitter yttrium-90, have EU approval for use in inoperable liver cancer and have been utilised in published phase I-II studies [9-11], which have demonstrated tolerability, high response rates and improved time to progression versus chemotherapy alone.

In contrast to surgical resection and thermal ablation (e.g. radiofrequency ablation or microwave), SIRT is not limited by the number or sites of liver metastases. The technique of SIRT involves an out-patient procedure in which a trans-femoral catheterisation is performed and, in the case of resin microspheres, approximately 40 to 80 million microspheres are injected into the arterial supply of the liver under fluoroscopic guidance. This microsphere infusion is secondary to a pre-treatment ‘work-up’ procedure: hepatic angiography with application of Technetium-99 macro-aggregated albumin in conjunction with gamma scintigraphy is used to predict microsphere distribution and also ascertain the extent of hepatopulmonary shunting. Whereas the normal liver receives the majority of its blood supply from the portal venous system, liver tumours obtain the majority of theirs from the hepatic artery. SIRT exploits this vascular phenomenon, and as a consequence ensures the deposition of the infused microspheres into the malignant microvasculature [7,8], delivering a high dose of radiation to tumour cells whilst relatively sparing the normal liver parenchyma. The preferential lodging of microspheres within tumour microvessels derived from the hepatic artery has been demonstrated in patients treated with SIRT [8].

With escalating costs associated with multiple chemotherapies combined with biologics, and controversies regarding sequencing and combination of drugs for optimal effect, the role of combining radiosensitisers with liver-directed therapies needs to be defined. Reflecting this, the National Institute of Clinical Excellence (NICE) Interventional Guidance have emphasised the need for research studies with outcome measures which include survival “to reduce the current uncertainty about the efficacy of the procedure”.

The FOXFIRE trial will test the hypothesis that the addition of SIRT to chemotherapy (OxMdG: oxaliplatin, 5-fluorouracil and folic acid) for patients with liver-only or liver-dominant mCRC will show superiority when compared to chemotherapy alone, in terms of efficacy, safety and cost-effectiveness. Importantly, since the greatest clinical benefit may be obtained from combining radiotherapy with radiosensitising chemotherapy, FOXFIRE will combine SIRT with radiosensitising oxaliplatin and 5-fluorouracil chemotherapy at doses defined clearly in an earlier phase I-II study [11].

## Methods/Design

The FOXFIRE trial will be conducted in accordance with the Declaration of Helsinki, and all participating centres must obtain the relevant approvals before patient enrolment. The FOXFIRE trial has been approved by the National Research Ethics Service Committee South Central – Berkshire (REC reference: 09/H0505/1). The inclusion and exclusion criteria for the FOXFIRE trial are summarised in Table 1.

**Table 1:** FOXFIRE Inclusion/Exclusion criteria

**Inclusion criteria**	**Exclusion criteria**
1. Histologically confirmed colorectal cancer (CRC) with liver-only or liver-dominant metastases not amenable to curative surgical resection confirmed at a Multi-disciplinary team (MDT) meeting	1. Liver metastases amenable to curative resection, unless limited EHD
2. Unequivocal & measurable CT evidence of liver metastases, not treatable by surgical resection or local ablation with curative intent at time of trial entry	2. Pregnant or breast-feeding
3. Age ≥ 18 years	3. Evidence of ascites, cirrhosis or portal hypertension
4. WHO performance status of 0–1	4. Main portal venous tumour involvement or thrombosis
5. Life expectancy > 3 months	5. Previous radiotherapy to upper abdomen or upper lumbar spine
6. Adequate haematological, renal and hepatic function	6. Other active malignancy within last 5 years [excluding colorectal cancer and other non-melanoma skin cancers]
7. Eligible for systemic chemotherapy as 1st line treatment for metastatic CRC	7. Non-malignant disease that would render patient ineligible at the discretion of the Investigator
8. Liver only/limited extra-hepatic disease (EHD): metastases in the lung must not be more than 5 in number and should be, in the opinion of either the local multi-disciplinary team (MDT) or following central review of scans arranged via the Trials Office, amenable to future definitive local therapy. In addition to lung metastases, a single site of other extra-hepatic disease is permitted (e.g. multiple lymph nodes in one lymph node region) after approval by the Trials Office.	8. Equivocal, immeasurable, or unevaluable liver metastases
9. Patients are permitted to have a primary colorectal tumour in situ, which should be potentially resectable following protocol therapy	9. Unequivocal evidence of bone metastasis
10. Suitable for all aspects of treatment determined by clinical assessment undertaken by Investigator	10. DLT associated with previous 5-FU or oxaliplatin chemotherapy
11. Using adequate contraception if pre-menopausal (male and female patients)	11. Previous chemotherapy for metastatic colorectal cancer [last dose of adjuvant chemotherapy for CRC administered ≥ 6 months pre-randomisation]
12. Willing & able to provide written informed consent	12. Peripheral neuropathy > CTCAE Grade 1

### Overview of trial design

**Figure 1:**
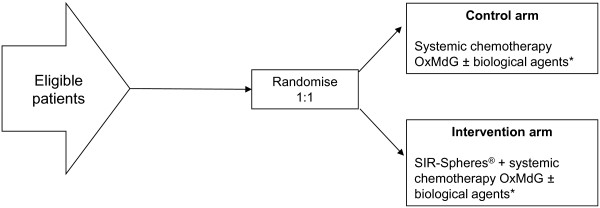
**Basic clinical trial schema for FOXFIRE clinical trial.** Dose modification of oxaliplatin in the Intervention Arm is described in the main text. *Biological agents (e.g. cetuximab or bevacizumab) are permitted from cycle 1 in the control arm or from cycle 7 in the intervention arm.

The FOXFIRE trial is a phase III, open label, multicentre, parallel two-arm, randomised controlled trial of OxMdG chemotherapy with or without the addition of interventional SIRT for patients with liver-only or liver-dominant mCRC. Patients will be recruited at 34 UK centres with 20 being specialist SIRT centres and the remaining being chemotherapy-only centres feeding into pre-specified SIRT centres. Prior to site activation, all SIRT centres will have treated at least three patients with SIRT to demonstrate that they have adequate training, support and mentoring in place to perform the treatment safely. Eligible patients are randomised 1:1 to receive either systemic chemotherapy with oxaliplatin, 5-fluorouracil and folic acid (OxMdG) or single-session whole liver SIRT + OxMdG (Figure 1).

### Randomisation and stratification

Following consent, eligible patients will be randomised 1:1 to OxMdG ± SIRT using a central computer system. Simple randomisation is used for the first 30 patients followed by minimisation with a random element (0.8) [12] and using the stratification factors: presence or absence of extra-hepatic metastases, extent of tumour involvement of the liver (≤25% or >25% tumour involvement determined by computed tomography [CT] scan), investigational centre, and intention to treat with a biological agent (introduced in March 2011 following a shift in the international standard treatment paradigm for these patients).

### Protocol treatment

Systemic chemotherapy must start within 28 days of randomisation. In the control arm, systematic chemotherapy with OxMdG consists of oxaliplatin (85 mg/m^2^ infusion over 2 hours), folinic acid (l-folinic acid 175 mg or d, l-folinic acid 350 mg infusion over 2 hours) and 5-FU (400 mg/m^2^ bolus followed by a 2400 mg/m^2^ continuous infusion over 46 hours). This cycle is then repeated every 14 days for 12 cycles. In the treatment arm, SIRT is administered on the third or fourth day of the second chemotherapy cycle. In addition, the same chemotherapy regimen is used except in cycles 2–4 when the oxaliplatin dose was reduced to 60 mg/m^2^ as this has been demonstrated as the maximum tolerated dose in an earlier phase I-II trial [11]. SIRT requires a hepatic arteriogram and a liver-to-lung breakthrough nuclear medicine scan to ensure suitability for receiving this procedure, and to plan the delivery of the SIR-spheres. A separate SIR-Spheres users’ manual details the technique for delivery of the SIR-Spheres. The prescribed activity of SIR-Spheres will be determined from the patient’s body surface area (BSA), the percentage tumour involvement, and the magnitude of liver-to-lung shunting. The dosing charts used are consistent with a similar contemporary study (SIRFLOX study protocol, submitted to *BMC Cancer*, March 2014).

The use of a licensed biological agent (e.g. cetuximab, bevacizumab) is permitted in this trial at the discretion of the treating investigator and at doses determined by local practice, but the intention to treat a patient with a biological agent should be declared at the time of randomisation. Intention to treat with a biological agent is a stratification factor in the trial. The biological agent can be added at any time during protocol chemotherapy for patients randomised to chemotherapy only, but, on account of a potential interaction with liver radiotherapy, it should not be delivered prior to cycle 7 for patients randomised to receive SIRT.

Once protocol treatment is completed, patients should be given the best available care based upon clinical assessment and patient preference. In both arms, if following treatment response the patient is deemed a candidate for surgical resection (assessed at 3 and 6 months after starting protocol treatment), and the patient undergoes surgical resection and/or complete ablation of their primary and metastatic cancer, protocol OxMdG chemotherapy should be continued as scheduled if appropriate.

### Outcome measures and definitions

The primary endpoint of the FOXFIRE trial is a comparison of OS between treatment arms. Secondary endpoints include safety, progression-free survival (PFS), liver-specific PFS, response and resection rate, time until next therapy, patient-reported outcomes (PROs) and cost-effectiveness. All patients will be assessed by the criteria.

OS is defined as the time from randomisation until death from any cause with patients censored at the last date alive if they are not known to have died by the end of the follow-up period. PFS is defined as the time from randomisation until disease progression (RECIST version 1.1 guidelines [13]) or death from any cause with censoring at the last date alive and progression-free. PFS and response rate will be determined from serial CT scans. Sample scans are being collected from each centre for central review as part of radiology quality assurance.

Liver-specific PFS is defined as for PFS but until documented progression in the liver. Adverse events (AEs) and Serious Adverse Events (SAEs) will be collected and rated according to Common Terminology Criteria for Adverse Events (CTCAE) v3.0 and the relationship to protocol therapy will be rated as none, unlikely, possible or probable.

PROs are assessed using the generic EORTC Quality of Life Questionnaire (QLQ-C30) and disease specific EORTC QLQ-LMC21 with pre-specified outcomes of global quality of life at 12 months and fatigue at cycle 4. Health economic outcomes will be based upon the quality of life measured by EQ-5D and collection of health economic data including costs of chemotherapy and SIRT (including the use of ancillary services and treatments), hospital visits, imaging, surgical procedures, and in-patient lengths of stay.

### Sample size calculation and statistical considerations

The entry criteria and trial design of FOXFIRE are sufficiently similar to the SIRFLOX clinical trial (SIRFLOX study protocol, submitted to *BMC Cancer*, March 2014) for the data from the two studies to be prospectively combined in a statistically valid way to improve the overall utility of both studies in answering the key research questions. The original sample size calculation required a total of 810 patients in order to observe 631 OS events with 80% power and two-sided 5% significance to detect a difference in median OS from 16 to 20 months (HR = 0.8), and allowing for a 5% drop-out/loss to follow-up and assuming 3 years recruitment and 2 years follow-up.

It was originally planned to recruit this number of patients from both FOXFIRE (n = 490) and SIRFLOX (n = 320) and carry out a combined analysis for OS. Following changes to the treatment paradigm in liver metastatic colorectal cancer SIRFLOX allowed the use of bevacizumab in suitable patients and the intention-to-treat with bevacizumab was included as a stratification factor. FOXFIRE also allowed appropriate treatment with a biological agent, including bevacizumab and cetuximab, adding intention-to-treat with a biological agent as a stratification factor. The use of biological agents will potentially prolong the PFS time and this has led to increasing the sample size for PFS in SIRFLOX. Thus, the overall sample size for the combined endpoint has been adjusted to take into account changes in the expected median OS for the control group. We therefore predict that 1022 patients will be required to observe the 631 events required to detect a hazard ratio of 0.8 with 80% power and two-sided 5% significance. This number of patients will include at least 320 patients recruited in FOXFIRE, 500 patients recruited in SIRFLOX and the additional patients being recruited into an extension study to SIRFLOX, called FOXFIRE-Global. It is anticipated that the required number of participants will have been recruited by October 2014.

All analyses will be conducted on an intention-to-treat (ITT) basis. The primary endpoint of OS will be analysed after all patients recruited into FOXFIRE and SIRFLOX have been followed up for at least 2 years and at least 631 deaths have been observed. Analysis will be by Kaplan-Meier survival curves and unadjusted log-rank test. The primary analysis will be a two-stage meta-analysis of individual patient data with the first stage consisting of within trial analyses and the second stage being a pooled analysis of the separate within trial analysis results. A secondary analysis will be undertaken using the one-stage approach also using individual patient data but for all patients, in order to investigate other aspects of the trial and covariates. Response and resection rates will be compared using test of proportions and the unadjusted log rank test will be used to compare time to event endpoints. PROs will be compared using ANCOVA adjusting for baseline values, with pre-specified variables compared using 5% (2-sided) significance and the other functional and symptom scales assessed using 1% (2-sided) significance.

Sensitivity analyses will be performed adjusting for minimisation and prognostic factors and by trial (FOXFIRE/SIRFLOX) in a multivariate analysis framework. This includes the impact of explanatory variables on various outcome measures of interest. Proportional hazards regression will be used to model time to event outcomes, multiple linear regression to model continuous outcomes (with appropriate transformations if necessary) such as the PROs, logistic regression to model binary outcomes (such as response) and ordered logistic regression for ordered categorical outcomes (such as toxicity grades).

### Planned interim analyses

The Data and Safety Monitoring Committee (DSMC) will review the results from several interim analyses using data from both the FOXFIRE and SIRFLOX trials, these include: analysis of toxicity and safety approximately 8 months after at least 80 patients are randomised (a minimum of 40 patients per trial); and analysis of toxicity and safety approximately 8 months after at least 300 patients are randomised (a minimum of 120 patients per trial). Further specifics of all analyses will be agreed with the DSMC and included in the statistical analysis plan.

## Discussion

In their procedure guidance published in July 2011, The UK National Institute for Health and Clinical Excellence (NICE) stated that the evidence on the efficacy of SIRT in chemotherapy-naïve patients was lacking and “Clinicians should offer eligible patients who have not been previously treated by chemotherapy entry into well-designed research studies such as the FOXFIRE trial (http://www.octo-oxford.org.uk/alltrials/trials/FOXFIRE).” FOXFIRE is the first clinical study of SIRT to be powered to address an overall survival endpoint and to include systematic evaluation of quality of life and healthcare economics.

As the first multi-centre, phase III clinical trial of SIRT in the UK, FOXFIRE has resulted in the establishment of a national infrastructure for the safe delivery of SIRT to patients with cancer, and “chemotherapy-only” centres who deliver FOXFIRE chemotherapy and refer their patients to another centre for the SIRT procedure. This pattern of specialist of SIRT centres and “chemotherapy-only” centres has improved equity of access to this clinical trial across the whole of the UK and is particularly important for interventional oncology trials to maintain adequate recruitment rates. Recruitment to FOXFIRE was one of the factors used by NHS England in deciding which 10 centres would be commissioned to deliver SIRT as part of the Commissioning through Evaluation programme for the 3^rd^ and subsequent line therapy of metastatic colorectal cancer [14].

SIRFLOX is a randomised multi-centre trial with participating centres in Australia, New Zealand, Europe and the USA, evaluating the addition of SIR-spheres to FOLFOX chemotherapy for patients with non-resectable liver metastases from primary colorectal carcinoma either with or without evidence of extra-hepatic disease. FOLFOX is equivalent in drug doses to OxMdG but uses a different delivery regimen with regard to the sequencing of drugs. Discussion between the trial management groups of the two trials led to the development of similar protocols and treatment paradigms in order to provide sufficient power for the prospective combined analysis of the two trials for overall survival. This will be carried out as a two-stage process: separate within trial analyses followed by a meta-analysis pooling these results. Additional analysis using the individual patient data will also be undertaken. A combined statistical analysis plan will be finalised prior to final OS datalock combining data from the two trials. This result will be in addition to the PFS being reported separately for SIRFLOX and FOXFIRE, anticipated in 2015 and 2016 respectively.

The need for robust quality of life information from patients having SIRT was emphasised by NICE in its guidance published in July 2011. To address this deficit in the published literature, FOXFIRE has incorporated 3 quality of life instruments at 4 timepoints, plus annually, as an important secondary endpoint of the clinical trial. In addition to these data, a bespoke health economics questionnaire is being collected at baseline and annually to provide information about potential health economic benefits for patients treated with SIRT.

FOXFIRE also includes translational substudies, including the collection of blood and tissue samples jointly with the New-EPOC clinical trial (University of Southampton, UK: ISRCTN22944367) under the combined Trans-EPOC award (separate grant from Cancer Research UK). The biobank being collected from FOXFIRE represents the only tissue collection worldwide of liver tissue post-radiotherapy and it represents a valuable resource for researchers to access via the Trans-EPOC trial management group. A substudy of Perfusion Liver CT in a subgroup of the FOXFIRE patients is also open to recruitment and is gathering data to study the role of this imaging modality in predicting response to chemotherapy and to SIRT (PERFORM, University of Oxford, UK: NCT01410760). These translational and radiological substudies will inform investigators how best to select patients for SIRT and how to assess response.

In summary, the FOXFIRE trial is testing the hypothesis that combining radiosensitising chemotherapy (OxMdG) with SIRT using yttrium-90 resin microspheres as a first-line treatment for liver-dominant metastatic CRC will improve clinical outcomes when compared to OxMdG chemotherapy alone.

## Abbreviations

BSA: Body surface area; CEA: Carcino-embryonic antigen; CRC: Colorectal cancer; CT: Computed tomography; 5-FU: 5-fluorouracil; HRQoL: Health-related quality of life; ITT: Intention-to-treat; MDT: Multi-disciplinary team; OS: Overall survival; OxMdG: Oxaliplatin and modified de Gramont (5-fluorouracil) chemotherapy; PFS: Progression-free survival; PRO: Patient-reported outcome; RE: Radio-embolisation (also known as SIRT); RECIST: Response evaluation criteria in solid tumours; SIRT: Selective internal radiation therapy.

## Competing interests

RAS has received research funding from Sirtex Medical Limited for the FOXFIRE clinical trial. Other authors have declared no competing interests.

## Authors’ contributions

RS and HSW conceived the FOXFIRE study, designed it with input from the NCRI Colorectal Clinical Study Group, submitted it for funding from the Bobby Moore Fund of Cancer Research UK, and drafted this manuscript. NK is the Trial Coordinator and manages the day-to-day running of the trial and drafted this manuscript. SBL and SD participated in the design and organisation of the study and provided statistical advice and drafted this manuscript. All authors read and approved the final draft of the manuscript for submission.

## Pre-publication history

The pre-publication history for this paper can be accessed here:

http://www.biomedcentral.com/1471-2407/14/497/prepub
